# CLASS-M: Adaptive stain separation-based contrastive learning with pseudo-labeling for histopathological image classification

**DOI:** 10.1016/j.media.2025.103711

**Published:** 2025-07-08

**Authors:** Bodong Zhang, Hamid Manoochehri, Man Minh Ho, Fahimeh Fooladgar, Yosep Chong, Beatrice S. Knudsen, Deepika Sirohi, Tolga Tasdizen

**Affiliations:** a Department of Electrical and Computer Engineering, University of Utah, Salt Lake City, UT, USA; b Scientific Computing and Imaging Institute, University of Utah, Salt Lake City, UT, USA; c Department of Electrical and Computer Engineering, University of British Columbia, Vancouver, BC, Canada; d Department of Hospital Pathology, College of Medicine, The Catholic University of Korea, Seoul, South Korea; e Department of Pathology, University of Utah, Salt Lake City, UT, USA

**Keywords:** Contrastive learning, Stain separation, Pseudo-labeling, Semi-supervised learning, Digital histopathological images

## Abstract

Histopathological image classification is an important task in medical image analysis. Recent approaches generally rely on weakly supervised learning due to the ease of acquiring case-level labels from pathology reports. However, patch-level classification is preferable in applications where only a limited number of cases are available or when local prediction accuracy is critical. On the other hand, acquiring extensive datasets with localized labels for training is not feasible. In this paper, we propose a semi-supervised patch-level histopathological image classification model, named CLASS-M, that does not require extensively labeled datasets. CLASS-M is formed by two main parts: a contrastive learning module that uses separated Hematoxylin images and Eosin images generated through an adaptive stain separation process, and a module with pseudo-labels using MixUp. We compare our model with other state-of-the-art models on two clear cell renal cell carcinoma datasets. We demonstrate that our CLASS-M model has the best performance on both datasets. Our code is available at github.com/BzhangURU/Paper_CLASS-M/tree/main.

## 1. Introduction

Digital histopathological image analysis plays a crucial role in disease diagnosis and treatment optimization. The most commonly used histopathological images are Hematoxylin & Eosin (H&E) stained whole slide images (WSIs) that help the differentiation of various tissue sample features (Fischer et al., 2008). H&E staining involves the use of two dyes, Hematoxylin (H) and Eosin (E), which selectively stain different components of the tissue samples. Hematoxylin stains the acidic components of the tissue samples, such as the cell nuclei, and has a blue-purple color, whereas Eosin stains the basic components of the tissue samples, such as the cytoplasm and extracellular matrix, and exhibits a pink color. In recent years, many efforts have been made towards the automatic analysis of H&E images.

With the rapid development of computing power and advanced algorithms, deep learning has been widely used in the field of digital histopathological image analysis for automating disease diagnosis and performing auxiliary image analysis (Chan et al., 2020; Litjens et al., 2017; Razzak et al., 2018; Sarvamangala and Kulkarni, 2022; Wang et al., 2022a). However, achieving a highly accurate model requires a substantial amount of training labels, demanding significant time and effort from human experts. Various strategies have been proposed to mitigate this challenge. Multiple instance learning (MIL), a specific type of weakly supervised learning, has become the accepted approach in histopathology because of the easy availability of case-level labels from medical reports. Self-supervised learning is also commonly used to train the backbone feature extractor network. Nevertheless, semi-supervised learning, despite its success in computer vision, remains a relatively less explored area in histopathology.

Another direction worthy of further investigation with deep learning models is kidney cancer. Kidney cancer ranks among the most prevalent cancers globally. It is estimated that around 76,080 new cases were diagnosed with cancers of the kidney and renal pelvis, and there are around 13,780 deaths resulting from it in the US in 2021 (Motzer et al., 2022). Clear cell renal cell carcinoma (ccRCC) stands out as the predominant subtype that dominates kidney cancer cases (Bian et al., 2022). Consequently, further research on cancer detection and classification using ccRCC images holds paramount importance for disease diagnosis and early patient treatment. In this paper, we experiment with two ccRCC datasets. Even though MIL approaches, requiring only slide-level annotations, are widely used in the histopathology field (Laleh et al., 2022; Li et al., 2023; Qian et al., 2022; Wang et al., 2022b), they require a large number of WSIs for effective training. For instance, the Utah ccRCC dataset we experiment with consists of only 49 WSIs. Additionally, MIL methods typically classify WSIs based on only the most discriminative patches, leading to poor patch-level accuracy despite demonstrating good slide-level accuracy. This can be a problem even for larger datasets such as The Cancer Genome Atlas Program (TCGA) ccRCC dataset we experiment with, which comprises 420 WSIs.

In our approach, pathologists draw approximate polygons at a low resolution to mark regions in a subset of WSIs and assign labels to those regions. We crop patches from inside these annotated regions to collect labeled samples for performing patch-level classification tasks. To fully utilize the WSIs, we also collect patches from outside the annotated regions and from WSIs that are not annotated to gather unlabeled samples. Consequently, we form a semi-supervised classification task based on the labeled and unlabeled patches. Notably, a patch-level classification model serves as a precise tool to expedite pathologists’ identification of cancerous regions within WSIs.

In this paper, we propose a new semi-supervised model for histopathological image classification: Contrastive Learning with Adaptive Stain Separation and MixUp (CLASS-M), where CLASS-M can also be understood as a model for CLASSifying Medical images. CLASS-M is an extension of Zhang et al. (2022a) presented in MICCAI 2022 Workshop on Medical Image Learning with Limited and Noisy Data with substantial modifications, a more thorough experimental analysis, and significant improvements in classification accuracy. A simple and effective novel unsupervised loss is formulated for contrastive learning between adaptively stain-separated Hematoxylin images and Eosin images. Additionally, considering the benefit of pseudo-labeling in state-of-the-art general semi-supervised learning methods (Sohn et al., 2020; Berthelot et al., 2019), pseudo-labeling with MixUp (Zhang et al., 2017) is adopted to provide further regularization via additional samples and pseudo-labels in training. The two methods benefit from different regularization effects, contrastive learning with multiple views vs. data augmentation, and hence can be combined to better utilize the information in both labeled and unlabeled samples and improve classification accuracy. Furthermore, instead of using a globally fixed stain separation matrix in Optical Density space as in Zhang et al. (2022a), a simple and efficient slide-by-slide stain separation based on Macenko et al. (2009) is applied to adapt to properties of individual WSIs. We not only perform image augmentations on Hematoxylin images and Eosin images, but also apply augmentations on original RGB images before the stain separation to strengthen the augmentation process. Finally, in this paper, we compare our method to multiple state-of-the-art semi-supervised and self-supervised learning models on the Utah ccRCC dataset and TCGA ccRCC dataset. We also conduct ablation studies to carefully analyze the contributions of different parts in our model.

In conclusion, the main contributions of our paper are as follows:

- We propose the CLASS-M model for semi-supervised learning. We apply our novel contrastive learning on Hematoxylin images and Eosin images after performing slide-level stain separation. We also use pseudo-labeling with MixUp to further improve classification accuracy.
- We provide the new Utah ccRCC dataset, which has 49 WSIs with patch-level labels, and patch-level labels for 150 WSIs from the TCGA ccRCC dataset. The Utah ccRCC dataset and its annotations will be made available upon transfer agreement. The annotations on the TCGA ccRCC dataset are made publicly available.
- We test various state-of-the-art semi-supervised learning and self-supervised learning methods on both ccRCC datasets and demonstrate that CLASS-M outperforms other state-of-the-art models.
- The code for our paper is available at github.com/BzhangURU/Paper_CLASS-M/tree/main

## 2. Related work

### 2.1. Weakly supervised learning

Weakly supervised learning (Kanavati et al., 2020; Oquab et al., 2015; Zhou, 2018) leverages slide or case-level labels to help guide the training process. A typical case of weakly supervised learning is MIL (Foulds and Frank, 2010; Herrera et al., 2016). Various MIL approaches, such as instance-based and embedding-based methods, have been proposed. The instance-based approach (Hou et al., 2016; Chikontwe et al., 2020) involves modeling a classifier at the instance level, and then aggregating the predicted instance labels to form bag label predictions. However, due to the noise in instance labels, the performance of this aggregation may be impacted. On the other hand, the embedding-based approach (Hashimoto et al., 2020; Li et al., 2021) first creates a bag representation from individual instance representations, followed by training a classifier on these bag representations. Research has shown that embedding-based approaches are generally more effective than instance-based methods. The attention-based MIL method, proposed in Ilse et al. (2018), suggests that instead of treating all patches within a bag equally, assigning varying importance scores to patches, particularly the more discriminative ones, is more effective. This method involves computing attention scores from instance representations to reflect the importance of respective patches and forming the bag representation through a weighted average of these instances. Xiong et al. (2023) proposed a hierarchical attention-guided MIL that effectively identifies important areas at different scales in WSIs. This method combines several attention techniques to form a comprehensive group representation. Furthermore, the recent Self-ViT-MIL approach by Gul et al. (2022), which combines self-supervised learning with Vision Transformers (ViTs) (Dosovitskiy et al., 2020) and MIL, has shown promising results, even in comparison to fully-supervised methods.

In digital histopathological image applications, MIL can be applied when only whole slide image-level labels are provided, but the specific regions that contribute to the labels are not given. Despite the convenience of annotation work, weakly supervised learning cannot deal with small datasets so well, as it needs an adequate amount of slides for learning.

### 2.2. Self-supervised learning

Self-supervised learning (SSL) (Jaiswal et al., 2020; Krishnan et al., 2022) extracts useful patterns from data itself without explicit labels provided by humans. Self-supervised learning allows models to first pre-train on a large unlabeled dataset, where effective feature representations can be learned. Examples of pre-training tasks include image inpainting, predicting rotations, and colorizing images. Then the final layers of the models can be optimized in specific downstream tasks, reducing the need for extensively labeled data. SimCLR (Chen et al., 2020a,b) presents a seminal contrastive learning to train feature representation, where augmented views of an image (positive samples) are minimized within that image while being maximized against augmented views of other images (negative samples). MoCo (Chen et al., 2020c, 2021; He et al., 2019, 2020, 2022) strengthens SSL with a dynamic dictionary, tapping into past batches for more negative samples, and a slowly updating momentum encoder for stable global representations. In contrast, Barlow Twins (Zbontar et al., 2021) omits negative samples, focusing on refining features by measuring cross-correlation between positive samples. BYOL (Grill et al., 2020) and DINO (Caron et al., 2021) utilize a teacher–student setup, in which the student refines its understanding by predicting the teacher’s representation, while the teacher network is updated through the slow-moving average of the student’s parameters. Clustering-based methods such as Deep Clustering (Caron et al., 2018) and SwAV (Caron et al., 2020) define pseudo-labels, sorting images into clusters, yielding great performance on downstream tasks.

In histopathology image analysis, self-supervised learning can be used to provide the backbone feature extraction module for MIL and patch-level classification models. Kang et al. (2023) benchmarked SSL methods on pathology datasets, showcasing consistent enhancements in histopathology tasks. CS-CO (Yang et al., 2022) also utilizes separate encoders for Hematoxylin images and Eosin images. Instead of contrastive loss between Hematoxylin images and Eosin images introduced in our work, CS-CO calculates a contrastive loss between different augmentations. In addition, CS-CO is a self-supervised learning method that requires encoders to generate a visual representation containing enough information to recover images in cross-stain prediction, whereas our semi-supervised CLASS-M only pursues shared latent features containing enough information to perform classification tasks. Although self-supervised learning benefits from unlabeled data, semi-supervised learning has the advantage that it allows both unlabeled data and labeled data to be trained at the same time and use the knowledge learned from labeled data to better utilize unlabeled data.

### 2.3. Semi-supervised learning

Semi-supervised learning (Chartsias et al., 2020; Ouali et al., 2020; Van Engelen and Hoos, 2020) involves training a model simultaneously with both labeled and unlabeled data to enhance training outcomes, which reduces reliance on a limited number of annotated samples. Semi-supervised learning finds applications when obtaining fully labeled data is prohibitively expensive or impractical. For digital histopathological image analysis, getting a large amount of labeled data requires great effort from well-trained experts, which is expensive and time-consuming. However, it is much easier to acquire unlabeled data such as unannotated WSIs. One type of semi-supervised learning is consistency regularization, which seeks agreement among the model predictions for the same input with different views, augmentations, or epochs. For example, temporal ensembling (Laine and Aila, 2016) aims to reach a consensus in the prediction of labels between current epochs and previous epochs. Sajjadi et al. (2016) tries to make the classifier’s prediction consistent across various transformations. More recent consistency regularization methods, such as FixMatch (Sohn et al., 2020) and MixMatch (Berthelot et al., 2019), have been proposed. FixMatch assigns pseudo-labels to unlabeled samples when the prediction confidence is high with weak augmentation of input data. Then these pseudo-labels are used for training with strong augmentation. MixMatch is another consistency regularization method where pairs of labeled/unlabeled input data are linearly combined to create a “mixup” of data. The label of newly created data is a weighted average of the original labels followed by a sharpening process. Inspired by self-supervised learning, contrastive learning has also been integrated into semi-supervised learning (Alonso et al., 2021; Chaitanya et al., 2023; Singh et al., 2021; Zhang et al., 2022a). Instead of seeking agreements in label predictions, contrastive learning focuses on the representation of positive and negative pairs. Positive pairs originate from the same inputs with different views or augmentations, while negative pairs involve different inputs. The method encourages a shorter distance between positive pairs and a longer distance between negative pairs in the feature representation space. In this paper, we propose a novel histopathology-specific contrastive learning based semi-supervised classification model.

## 3. Methods

### 3.1. An overview of CLASS-M

**Fig. 1.**
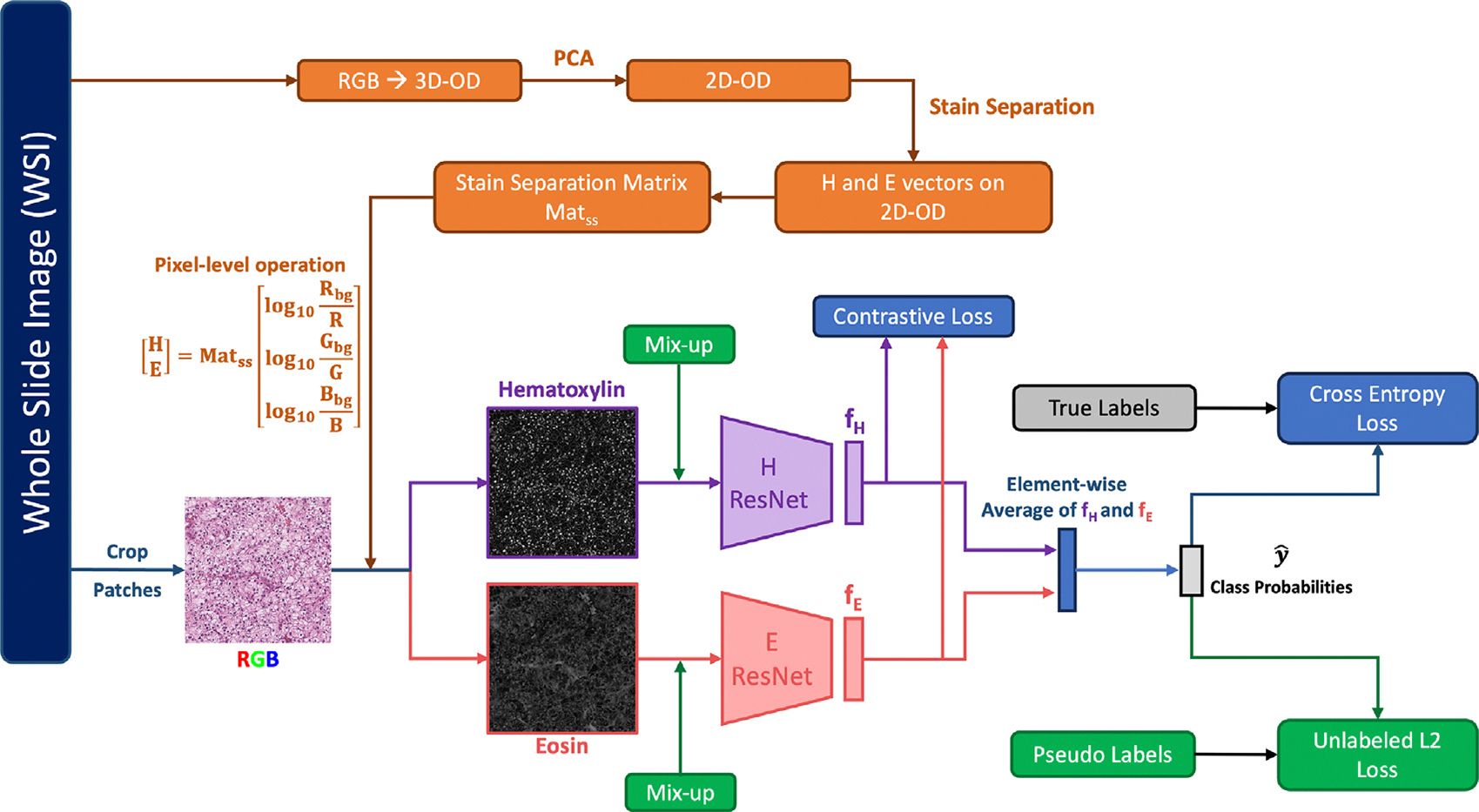
The block diagram of our Contrastive Learning with Adaptive Stain Separation and MixUp (CLASS-M) for semi-supervised histopathological image classification. The orange part shows adaptive stain separation, where OD means Optical Density space. The green part shows mixup on both labeled and unlabeled samples.

Fig. 1 shows the workflow of CLASS-M. As shown in the orange part of Fig. 1, slide-level stain separation is applied for generating Hematoxylin images and Eosin images from original RGB images. The original RGB images, Hematoxylin images, and Eosin images are all augmented during training to improve the robustness of the CLASS-M model. The model takes Hematoxylin images and Eosin images as input pairs to form different views of input data. The purple and pink boxes in Fig. 1 illustrate the H and E ResNet (He et al., 2016) encoders that have the same architecture but separate parameters to generate latent features $f_{H}$ and $f_{E}$. A contrastive loss is proposed for shared latent feature space between H channel and E channel to increase the similarity between $f_{H}$ and $f_{E}$. We take the average of features $f_{H}$ and $f_{E}$ and pass it to a linear+softmax layer to predict the labels. For labeled samples in training, the cross-entropy loss is introduced to measure the difference between predictions and true labels. Moreover, a challenge in semi-supervised learning in general is the limited number of labeled samples. Pseudo-labeling on unlabeled samples has been widely used in many state-of-the-art semi-supervised learning methods (Berthelot et al., 2019; Sohn et al., 2020; Zhang et al., 2021). In MixMatch (Berthelot et al., 2019), after pseudo-labeling on original samples, the model further uses MixUp (Zhang et al., 2017) to introduce virtual samples by linear interpolation of two random samples. Inspired by those ideas and to fully utilize unlabeled samples, we first provide pseudo-labels to unlabeled samples, and then add MixUp on both labeled and unlabeled samples to create virtual samples. The labels of the generated samples after MixUp are set to the weighted averages of original labels/pseudo-labels with a sharpening process. The green part in Fig. 1 shows the workflow of this process for pseudo-labeling. We introduce different parts of the CLASS-M model in detail in the following subsections.

### 3.2. Adaptive stain separation

Stain separation is introduced to separate different types of stains present in histological images (Pérez-Bueno et al., 2022; Vasiljević et al., 2021; Zhang et al., 2022b), such as separating Hematoxylin and Eosin (H&E) images into Hematoxylin images and Eosin images. The process has many challenges due to the variation of stains caused by different manufacturers, storage conditions, and staining procedures. In our model, a simple and unsupervised method for adaptive stain separation (Macenko et al., 2009) is applied.

First, the RGB pixel values from slide images are transformed into 3D-Optical Density (3D-OD) space, where stain components are formed by linear combinations. The corresponding Beer–Lambert law (Beer, 1852; Lambert, 1760) for this operation is below:

(1) $$OD_{C}={log}_{10}\frac{I_{0,C}}{I_{C}}$$

The letter C represents a specific channel, such as a Red, Green, or Blue channel. The value $I_{0,C}$ refers to the intensity of light before passing through the specimen, which is background intensity like 255, and the value $I_{C}$ is the intensity of light after passing through the specimen. We can treat the OD value as a measurement of the absorption of light on different channels. Zero values in all channels on OD space correspond to cases of pure white background where there is no tissue absorbing light on that pixel. For each RGB pixel, we use a 3 × 1 vector $V_{OD}={\left(OD_{R},OD_{G},OD_{B}\right)}^{⊤}$ to show the values on OD space.

**Fig. 2.**
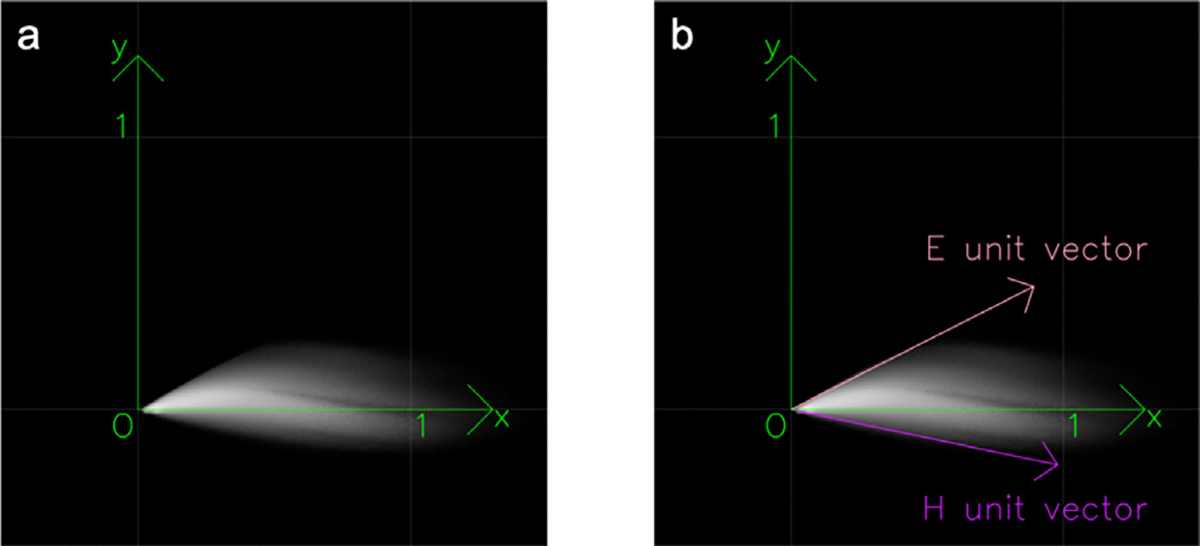
Mapping pixels onto the 2D OD space.

Then, dimensionality reduction from 3D-OD to 2D-OD is applied. In an ideal situation of H&E staining, $V_{OD}$ is a linear combination of fixed Hematoxylin and Eosin unit vectors: $V_{OD}=\alpha_{H}V_{H}+\alpha_{E}V_{E}$, where $V_{H}$ and $V_{E}$ are 3 × 1 unit vectors on 3D OD space. Therefore, all OD vectors should approximately fall into the same 2D plane for all pixels in one image. We use Principal Component Analysis (PCA) on 3D OD space to find the 2D plane formed by two covariance matrix’s eigenvectors with the largest two eigenvalues. Fig. 2.a is an example of the distribution of OD vectors that map onto the 2D plane. The brightness shows the density of pixels. The x-axis is the direction of the eigenvector with the largest eigenvalue. The y-axis is the direction of the eigenvector with the second-largest eigenvalue. The third dimension can be treated as a residual part and abandoned. The x and y axes are orthogonal because the eigenvectors of the covariance matrix are orthogonal to each other.

The third step is to find stain vectors $V_{H}$ and $V_{E}$. Macenko et al. (2009) assumes that pixel samples in OD space exist between the two stain vectors, considering that each component should be non-negative. An example of $V_{H}$ and $V_{E}$ is shown in Fig. 2.b. The positive directions of the x-axis and y-axis were carefully chosen such that the H unit vector falls into quadrant IV and the E unit vector falls into quadrant I.

After acquiring unit vectors $V_{H}$ and $V_{E}$, we are able to reconstruct the stain separation matrix. In the previous PCA process, we computed three unit eigenvectors $V_{x},\;V_{y},\;V_{\mathrm{Residual}}$, which are orthogonal to each other and ordered by their eigenvalues from maximum to minimum. The $V_{H}$ and $V_{E}$ on x-y 2D OD space can be written as $V_{H}=cos\;\theta_{H}V_{x}+sin\;\theta_{H}V_{y},\;V_{E}=cos\;\theta_{E}V_{x}+sin\;\theta_{E}V_{y}$. The transformation matrix from (H, E, Residual) to RGB on OD space is built as

(2) $${\mathrm{Mat}}_{HERes\to RGB}=\left[V_{H},V_{E},V_{\mathrm{Residual}}\right]$$

Therefore:

(3) $${\left(OD_{R},OD_{G},OD_{B}\right)}^{⊤}={\mathrm{Mat}}_{\mathrm{HERes}\to \text{RGB}}\times {\left(\alpha_{H},\alpha_{E},\alpha_{\mathrm{Residual}}\right)}^{⊤}$$

If we define ${\mathrm{Mat}}_{\mathrm{RGB}\to \mathrm{HERes}}$ as the inverse matrix, then:

(4) $${\left(\alpha_{H},\alpha_{E},\alpha_{\mathrm{Residual}}\right)}^{⊤}={\mathrm{Mat}}_{\mathrm{RGB}\to \mathrm{HERes}}\times {\left(OD_{R},OD_{G},OD_{B}\right)}^{⊤}$$

After applying the formula to all pixels in an image, the $\alpha_{H}$ forms the Hematoxylin image, and $\alpha_{E}$ forms the Eosin image. Finally, we normalize the images using their maximum intensity values.

The main advantages of this stain separation method are simplicity and efficiency. We are able to perform it without the need of model training or complex calculations. Moreover, finding optimum H vectors and E vectors helps to handle stain variations. The final normalization based on the calculated maximum value further mitigates the effect of variations on the brightness and strength of stains.

### 3.3. Augmentations on original RGB, Hematoxylin, and Eosin images

We perform color-jittering augmentations on brightness, contrast, and saturation of original images, followed by image augmentations on Hematoxylin images and Eosin images, including random crops, random rotations, and random flips. Independent jittering of brightness on Hematoxylin images and Eosin images is also applied. The extra augmentations on the original RGB images provide increased diversity in the training samples and help learn more general features.

### 3.4. Contrastive learning

Inputs from two different views are usually adopted in co-training. The general presumptions of co-training are that each input view carries sufficient information for the task and the views should be highly independent of each other to achieve good results. Our preliminary work (Zhang et al., 2022a) has demonstrated that Hematoxylin and Eosin channels fulfill these requirements in contrastive co-training.

**Fig. 3.**
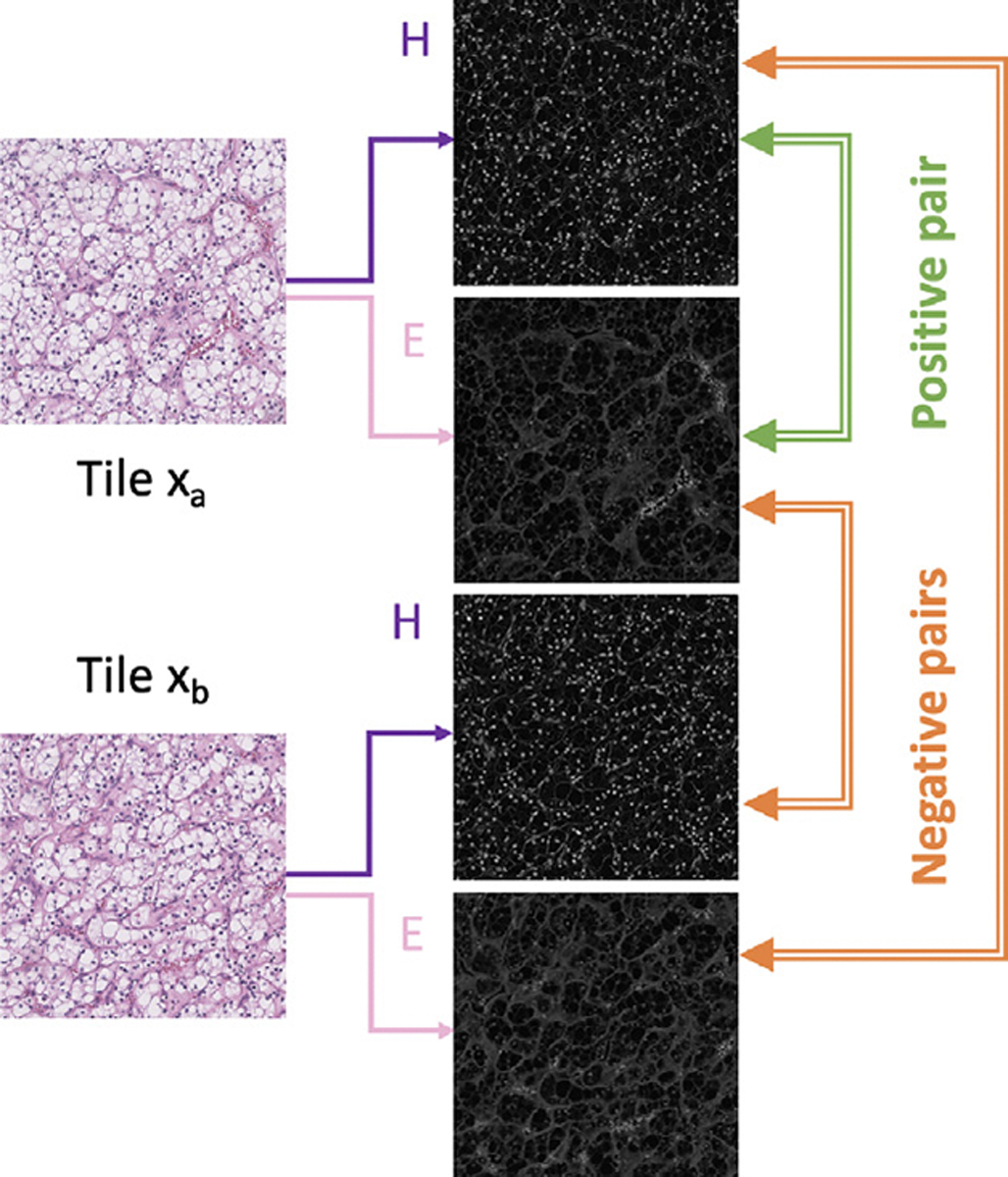
Contrastive learning on Hematoxyling images and Eosin images.

We use Triplet loss (Schroff et al., 2015) as the base of our contrastive loss. Let $x_{H}$ and $x_{E}$ denote the corresponding Hematoxylin channel image and Eosin channel image of the original image $x,\;f_{H}\left(x_{H}\right)$ and $f_{E}\left(x_{E}\right)$ denote the output features after the two ResNet models that take sample $x_{H}$ and $x_{E}$ as input separately. Then the contrastive loss function on sample $x_{i}$ is written as

(5) $${ℒ}_{ct}\left(x_{i}\right)=\;\;max\left({\left\|f_{H}\left(x_{i,H}\right)-f_{E}\left(x_{i,E}\right)\right\|}_{2}-{\left\|f_{H}\left(x_{i,H}\right)-f_{E}\left(x_{k,E}\right)\right\|}_{2}+m,0\right)$$

where $x_{k,E}$ represents the Eosin channel image of another random sample $x_{k},\;\|a{\|}_{2}$ denotes the L2 norm of vector a, and m serves as a margin hyperparameter. This contrastive loss term forms a positive pair whose $x_{H}$ and $x_{E}$ originate from the same sample and a negative pair whose $x_{H}$ and $x_{E}$ come from different samples. Fig. 3 shows an example of how the positive pairs and the negative pairs are formed.

During training, the H and E features from the same sample are pulled closer in the shared latent feature space, while the features from different samples are pushed further away. The Triplet loss encourages a balance between pulling positive samples closer and pushing negative samples further away. The mechanism of clipping some loss values to 0 ensures that the model focuses on learning from hard examples where the negative pairs are not significantly farther apart than the positive pairs. Additionally, zero loss for well-separated embeddings helps prevent overfitting by avoiding excessive optimization of already well-structured representations.

### 3.5. Mixup

MixUp (Zhang et al., 2017) is applied on both labeled and unlabeled samples. First, we assign pseudo-labels to unlabeled samples. Assuming $x_{i}$ is an original unlabeled sample from unlabeled training set U, we augment it K times and get the average of softmax predictions from the model. The result is sharpened to lower the entropy of label distribution using

(6) $$\bar{y_{i}}=\frac{1}{K}\sum_{1\leq k\leq K}P_{\mathrm{model}}\left(x_{i,k}\right),x_{i}\in U$$

(7) $$y_{i}=sharpen\left({\overset{‾}{y}}_{i},T\right),\;where\;sharpen{\left(y,T\right)}_{\left(c\right)}=\frac{{y_{\left(c\right)}}^{\frac{1}{T}}}{\sum_{j=1}^{C}{y_{\left(j\right)}}^{\frac{1}{T}}}$$

where $x_{i,k}$ iz the *k*th augmentation of sample $x_{i},\;T$ is a temperature hyperparameter to control the sharpness, $y_{\left(c\right)}$ is the c th element in one-hot label encoding y,C is the number of classes, and $y_{i}$ is the virtual one-hot label encoding of sample $x_{i}$.

We follow the standard procedure of MixUp to mix samples. Let $x_{i}$ and $x_{j}$ be two random samples from labeled training set L or unlabeled training set U, whose one-hot label encodings are $y_{i}$ and $y_{j}$. Let λ denote a random number from Beta distribution Beta(α,α), and set $\lambda^{\prime }=max(\lambda ,1-\lambda )$. Then the new mixed sample and its label are as follows:

(8) $$x_{i}^{\prime }=\lambda^{\prime }x_{i}+(1-\lambda^{\prime })x_{j}$$

(9) $$y_{i}^{\prime }=\lambda^{\prime }y_{i}+(1-\lambda^{\prime })y_{j}$$

After MixUp, we define $x_{i}^{\prime }$ as a labeled sample if $x_{i}$ is from labeled set to form new labeled set $L^{\prime }$, the remaining samples form new unlabeled set $U^{\prime }$ with virtual labels. Since $\lambda^{\prime }$ is random, sample diversity is greatly increased.

With MixUp, the contrastive loss term is still the same as before except that the inputs are changed to virtual MixUp samples. The new Hematoxylin images and Eosin images from virtual MixUp sample $x_{i}^{\prime }$ are defined as

(10) $$x_{i,H}^{\prime }=\lambda^{\prime }x_{i,H}+\left(1-\lambda^{\prime }\right)x_{j,H}$$

(11) $$x_{i,E}^{\prime }=\lambda^{\prime }x_{i,E}+\left(1-\lambda^{\prime }\right)x_{j,E}$$

where $x_{i,H}$ and $x_{i,E}$ denote the corresponding Hematoxylin channel image and Eosin channel image of the original image $x_{i}$, while $x_{j,H}$ and $x_{j,E}$ correspond to another random original image $x_{j}$. The contrastive loss on virtual sample $x_{i}^{\prime }$ with the application of MixUp is written as

(12) $${ℒ}_{ct}\left(x_{i}^{\prime }\right)=\;max\left({\left\|f_{H}(x_{i,H}^{\prime })-f_{E}(x_{i,E}^{\prime })\right\|}_{2}-{\left\|f_{H}(x_{i,H}^{\prime })-f_{E}(x_{k,E}^{\prime })\right\|}_{2}+m,0\right)$$

where $x_{k,E}^{\prime }$ comes from the Eosin channel image of another random virtual MixUp sample. Similarly, we define a positive pair in contrastive learning if $x_{H}^{\prime }$ and $x_{E}^{\prime }$ come from the same virtual MixUp sample, and a negative pair if $x_{H}^{\prime }$ and $x_{E}^{\prime }$ come from different virtual MixUp samples.

### 3.6. Loss function

The total loss is formed by the summation of losses from label prediction and contrastive learning. Inspired by MixMatch (Berthelot et al., 2019), we use cross-entropy loss in labeled set $L^{\prime }$ and squared $L_{2}$ loss between predictions and virtual labels in unlabeled set $U^{\prime }$ considering squared $L_{2}$ loss is less sensitive to incorrect predictions. The total batch loss can be written as

(13) $$ℒ=\sum_{x_{i}\in L^{\prime }}\frac{y_{i}\;log\;{\overset{ˆ}{y}}_{i}}{\left|L^{\prime }\right|}+\lambda_{U^{\prime }}\sum_{x_{i}\in U^{\prime }}\frac{{\left\|y_{i}\;-\;{\overset{ˆ}{y}}_{i}\right\|}_{2}^{2}}{C\left|U^{\prime }\right|}+\lambda_{C}\sum_{x_{i}\in L^{\prime }\cup U^{\prime }}{ℒ}_{c.t.}\left(x_{i}\right)$$

$y_{i}$ is one-hot encoding of labels (in $L^{\prime }$) or virtual labels (in $U^{\prime }),\;{\overset{ˆ}{y}}_{j}$ is the label prediction from the model, C is the number of classes, $\left|L^{\prime }\right|$ and $\left|U^{\prime }\right|$ are the size of labeled set and unlabeled set in a batch. $\lambda_{U^{\prime }}$ and $\lambda_{C}$ are hyperparameters that control the weights of squared $L_{2}$ loss and contrastive learning loss.

## 4. Experiments

### 4.1. Datasets

To evaluate our method, we applied CLASS-M on the Utah ccRCC dataset and TCGA ccRCC dataset separately, and compared results with other semi-supervised and self-supervised classification methods.

**Fig. 4.**
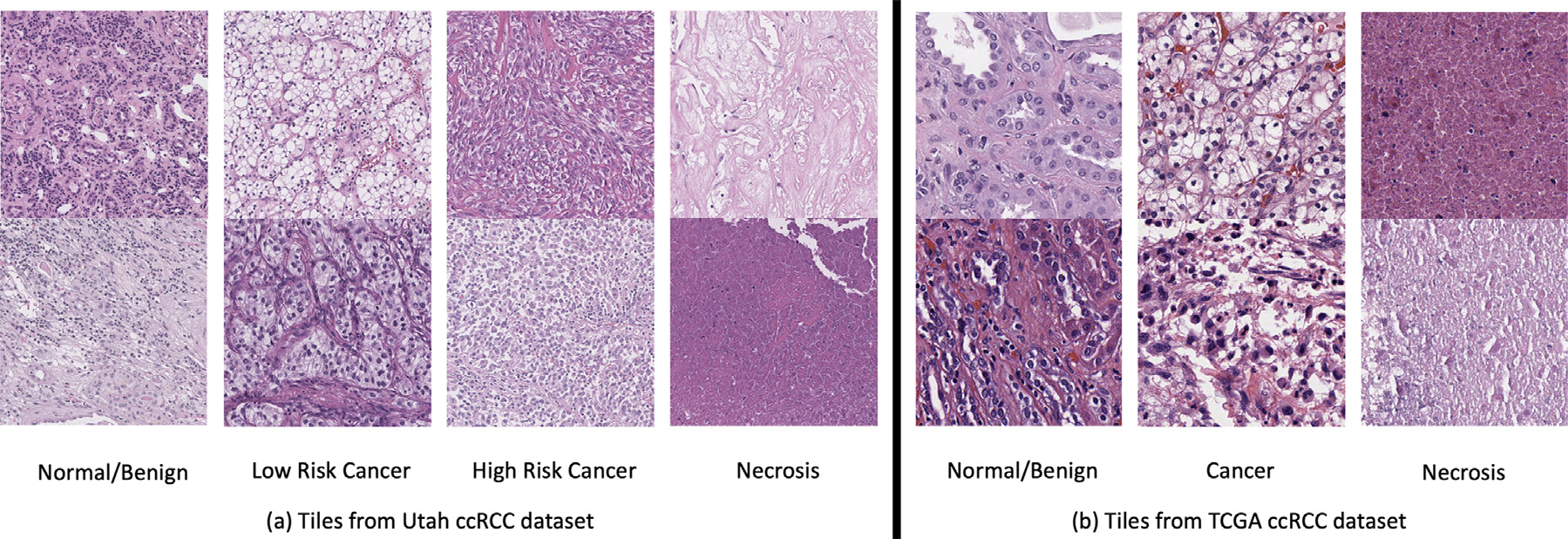
Examples of tiles with the size of 400 × 400 from (a) Utah ccRCC dataset (in 10X) and (b) TCGA ccRCC dataset (in 20X).

In the Utah ccRCC dataset, there are 49 WSIs from different patients. First, a pathologist drew polygons inside WSIs to mark areas with certain growth pattern labels, which were subsequently verified by another pathologist. We randomly split the WSIs into 32, 10, and 7 WSIs for training, validation, and test set. The WSIs were then cropped into 400 × 400 tiles at 10X resolution with stride set to 200 pixels inside each labeled polygon. The same process was applied to crop tiles outside polygons in the 32 training WSIs to collect unlabeled tiles to form a semi-supervised learning task for a 4-class classification. In detail, there are 28 497, 2044, 2522, and 4115 tiles in the category of Normal/Benign, Low risk cancer, High risk cancer, and Necrosis, respectively, in the labeled training set extracted from polygons in 32 WSIs. Additionally, there are 171,113 unlabeled training tiles, 5472, 416, 334, and 2495 tiles, respectively, for each category in the validation set, and 7263, 598, 389, and 924 tiles, respectively, for each category in the test set. Tiles with predominantly background areas were removed, along with those tainted by ink. Tile examples can be seen in Fig. 4(a).^2^

For the TCGA ccRCC dataset, we have in total 420 WSIs from different patients. 150 of them were labeled by a pathologist through drawing polygons with annotations and verified by a second pathologist. The remaining 270 WSIs were treated unlabeled. If pathologists had disagreements or concerns about labels of polygons, then those annotations were abandoned. The resolution we trained on is 20X to have more diversity and build a relatively larger dataset compared to the Utah ccRCC dataset. To make it a more challenging task and show the effectiveness of semi-supervised learning, we split the 150 labeled WSIs into 30, 60, and 60 WSIs for the training, validation, and test set. The tile cropping process was the same as in the Utah ccRCC dataset except for different strides. We chose 200 pixels as stride for the labeled training set, but 400 pixels for the validation and test set, considering they contain a large number of tiles. We cropped foreground tiles outside polygons in the 30 labeled training WSIs, as well as foreground tiles across the 270 unlabeled WSIs to form unlabeled training samples by setting the cropping stride to 400 pixels. The cropped tiles maintained 400 × 400 size at all times. We split the labeled tiles into 3 categories: Normal/Benign, Cancer, and Necrosis, to perform 3-class semi-supervised classification tasks. Tile examples can be seen in Fig. 4(b). In summary, there are 84 578, 180 471, and 7932 labeled training tiles, respectively, in the category of Normal/Benign, Cancer, and Necrosis, as well as 19 638, 79 382, 1301 validation tiles, and 15 323, 62 565, 6168 test tiles, respectively, for each category. The number of unlabeled training tiles is 1,373,684. The TCGA ccRCC dataset is a publicly available dataset, and we have also made our annotations for the 150 WSIs publicly accessible. The tiles described above can be easily generated through our code by reading WSIs and annotations as inputs.

### 4.2. Adaptive stain separation

In experiments, we maintained slide-by-slide level adaptive stain separation before the CLASS-M model training based on the following considerations: The stain styles vary across different WSIs due to different conditions. However, within one WSI, most conditions, such as stain manufacturer, storage condition, and staining procedure, remain consistent. On the other hand, if adaptive stain separation is applied patch-by-patch, there are not enough pixel samples inside each patch to obtain robust stain separation results. For example, in Fig. 4(a), the top image in the Necrosis category has very limited Hematoxylin portion, which makes the stain separation less trustful at patch-by-patch level.

As described in Macenko et al. (2009), in the step of finding stain vectors $V_{H}$ and $V_{E}$, considering the fact that noise cannot be fully avoided, we allowed 1% of pixel samples to be outside of $V_{H}$ and $V_{E}$, respectively. Moreover, pixel samples that are within a distance of 0.1 from the origin were not taken into account as they contain less stain and are more easily influenced by noise.

In the final step of further normalizing Hematoxylin images and Eosin images, the 99th percentile of intensity values was used as an approximation of maximum value. We normalized it to 0.5 and clipped to 1.0 for any numbers larger than 1.0 after normalization.

### 4.3. Experiment settings

For a fair comparison, we used ImageNet pre-trained ResNet18 model (He et al., 2016) as an initialization for all Convolutional Neural Network (CNN) models in semi-supervised learning. We compared our CLASS-M model with other state-of-the-art semi-supervised learning models, including FixMatch (Sohn et al., 2020) and MixMatch (Berthelot et al., 2019). In CLASS-M model training, we performed jittering of brightness, contrast, and saturation on original RGB images, as well as image augmentations on H images and E images after stain separation, such as random rotation, random crop to 256 × 256, random flip, and jittering of brightness. The image augmentations on H images and E images are independent, for example, they could have different rotation angles in random rotation. Since H images and E images are one-channel images, we summed the pre-trained weights on the first layer of ResNet18 as initialization weights.

We used Root Mean Squared Propagation optimization method with a decaying learning rate. A batch size of 64 was chosen for all classification experiments. A balanced sampler was used to address the imbalance in the number of labels in the training set. In experiments with the Utah ccRCC dataset, we chose to have 32 labeled samples in each batch, with 8 samples for each category, while the remaining 32 samples were from unlabeled samples. In experiments with the TCGA ccRCC dataset, we chose to have 33 labeled samples in each batch, with 11 samples for each category, and the remaining 31 samples would be unlabeled. In validation and test, we first calculated classification accuracies for each category and then averaged them to get balanced validation accuracy and test accuracy. This approach ensures that each category holds equal importance, irrespective of the actual number of tiles for each category. The hyperparameters were fine-tuned for all models to get the best validation accuracy, and then the test accuracy on that hyperparameter setting was reported as the final performance. We found that most hyperparameters did not need to be changed between the Utah ccRCC dataset and the TCGA ccRCC dataset. We chose a decaying learning rate with initial value set to 10^−4^. We set the number of augmentation times K to 2, temperature T to 0.5, α to 2, margin m in contrastive loss to 37, and unlabeled $L_{2}$ loss weight to 7.5 for both datasets. We set contrastive loss weight to 0.1 for experiments on the Utah ccRCC dataset, and contrastive loss weight to 0.001 for the TCGA ccRCC dataset. For each epoch, we ran 1000 iterations and checked validation accuracy once. The total epochs were large enough to ensure convergence. The training was stopped if the best validation accuracy was no longer updated for more than 100 epochs. Each experiment was repeated three times to obtain the average test accuracy and standard deviation.

The experiment platform is Python 3.7.11, Pytorch 1.9.0, torchvision 0.10.0, and CUDA 10.2. The GPUs we used are NVIDIA TITAN RTX. During CLASS-M model training, it usually took 13 GB GPU memory and 12 min for each training epoch.

### 4.4. Comparison with baseline and other semi-supervised models

We tested our CLASS-M model along with other state-of-the-art semi-supervised models, including FixMatch and MixMatch, using the Utah ccRCC dataset and TCGA ccRCC datasets. CLASS-M without pseudo-labeling using MixUp, which we name CLASS, was tested as a reference. The baseline ResNet18 (He et al., 2016) and Vision Transformer (ViT) (Dosovitskiy et al., 2020) were also tested where only labeled samples were used in training after initialization with weights pre-trained on ImageNet.

As shown in Table 1, the CLASS-M model outperforms not only the supervised baselines, but also other state-of-the-art models by a large margin on both datasets. We achieved a test accuracy of 95.35 ± 0.46% on the Utah ccRCC dataset, and 92.13 ± 0.89% on the TCGA ccRCC dataset. The ResNet18 baseline results from Table 1 indicate that TCGA ccRCC dataset presents a harder challenge. One possible reason is that the samples from the Utah ccRCC dataset have less variation in the staining procedure, and the storage conditions are uniform. However, on the TCGA ccRCC dataset, slides come from different institutions, leading to more variation in the quality of slides. Additionally, the Necrosis tiles on the TCGA training set only come from 4 WSIs, which causes more difficulties in learning robust features for this class. As shown in the recall section of Table 5 in Appendix A, pseudo-labeling with MixUp raised the Necrosis’s test accuracy from 60.47% to 86.65% on the TCGA ccRCC dataset, demonstrating that MixUp is especially effective on classes with very limited number of samples. In conclusion, our CLASS-M model achieves the highest accuracy results on a variety of tasks. We provide recall, precision, and F-score for each run, with details available in Appendix A. According to the recall results in Appendix A, in the Utah ccRCC dataset, semi-supervised models adopting the pseudo-labeling method have some limitations in predicting Normal/Benign patches compared to methods not using pseudo-labeling, like our CLASS model. Since Normal/Benign patches are dominant in the Utah ccRCC dataset, many were misclassified into other categories, resulting in low precision for those categories. Nonetheless, among the models utilizing pseudo-labels, our CLASS-M model consistently outperforms FixMatch and MixMatch in average recall, precision, and F-score across both datasets. After obtaining the CLASS-M models, we ran the models on WSIs from the test set and generated prediction heatmaps to better visualize patch-level predictions. The results can be found in Appendix B.

### 4.5. Comparison with self-supervised learning models

To compare with the self-supervised learning approaches, we used both labeled and unlabeled training samples to pre-train self-supervised learning models on the Utah ccRCC dataset and TCGA ccRCC dataset separately. After pre-training, the encoder was frozen, and a fully connected classification network was appended. The final fully connected classification layer was trained only on the labeled training samples. We selected several state-of-the-art self-supervised learning models in our experiments, including Barlow Twins (Zbontar et al., 2021), SwAV (Caron et al., 2020), MoCo v3 (Chen et al., 2021), and ViT-DINO (Caron et al., 2021), given their extensive use in histopathology (Kang et al., 2023). We used their default settings in the pre-training procedures and fine-tuned the downstream classification process. As shown in Table 1, our proposed models, CLASS and CLASS-M, outperform those self-supervised learning models on both ccRCC datasets. The recall, precision and F-score of the self-supervised learning models can also be found in Appendix A. The disadvantage of the semi-supervised approach compared to self-supervised learning is the loss of generality for the encoder, which must be re-trained for each new task. However, our results demonstrate that a well-formulated semi-supervised learning approach can have accuracy advantages over a self-supervised pre-training approach due to end-to-end learning with labeled and unlabeled samples simultaneously. Therefore, if accuracy is of paramount importance, semi-supervised learning may be preferred over self-supervised learning.

### 4.6. Ablation studies

We conducted ablation studies to further analyze our model and identify the components contributing to the classification results. One of the critical parts of our model is the H/E contrastive loss which provides a regularization term and helps to generate representative features. As shown in Table 2, removing the contrastive loss term led to a significant drop in the test accuracy. On the Utah ccRCC dataset, the test accuracy dropped from 95.35% to 90.92% for the CLASS-M model and from 94.92% to 84.57% for the CLASS model. On the TCGA ccRCC dataset, the test accuracy declined from 92.13% to 89.70% for the CLASS-M model and from 83.06% to 74.46% for the CLASS model.

Choosing Hematoxylin and Eosin channels as two views also plays a crucial role. Instead of choosing Hematoxylin and Eosin as two channels, we experimented with directly selecting two channels from Red, Green, and Blue channels in RGB images to form two views in the CLASS model. As a result, we saw large reductions in test accuracies. This result is expected due to the independence requirement in co-training which Hematoxylin and Eosin fulfill to a large extent, but Red/Green/Blue channels do not. In addition, splitting the original RGB images into Hematoxylin and Eosin channels is motivated by the pathology point of view and the true nature of H&E images.

The effect of adaptive stain separation was also verified. We used a globally fixed stain separation matrix to replace adaptive stain separation. The CLASS-M model’s test accuracy dropped from 95.35% to 93.97% on the Utah ccRCC dataset and dropped from 92.13% to 90.97% on the TCGA ccRCC dataset. We further did an ablation study on the augmentations on original RGB images. The augmentations proved beneficial and improved classification accuracy from 94.41% to 95.35% on the Utah ccRCC dataset and from 91.21% to 92.13% on the TCGA ccRCC dataset for the CLASS-M model. We finally analyzed the role of pseudo-labeling using MixUp. Table 2 shows that, by comparing CLASS-M with CLASS, we saw a substantial improvement by adding MixUp augmentation, especially on the TCGA ccRCC dataset.

### 4.7. Additional studies on contrastive loss design

Given that contrastive learning on the Hematoxylin channels and Eosin channels is a newly proposed idea, we also conducted extended experiments on the structural choices of contrastive loss functions to examine how different designs of these loss functions influence performance.

In the Triplet loss used as the backbone of our contrastive learning, the margin hyperparameter plays a crucial role in determining the minimum required separation between negative pairs and positive pairs. A large margin forces the negative samples to be pushed further away from the anchor compared to the positive samples. But if the margin is too large, the model may try to enforce unnecessary separation even for triplets that are already well-separated, leading to overfitting, poor generalization, or difficulties for the model to converge. On the other hand, a small margin results in less stringent constraints and allows the model to focus on hard examples. However, if the margin is too small, it may cause underfitting and the learned embeddings may not adequately separate the positive and negative pairs, leading to poor representation quality.

To further investigate the impact of the margin hyperparameter, we conducted experiments on CLASS-M using different margin values on both the Utah ccRCC dataset and the TCGA ccRCC dataset. As shown in Table 3, the optimum margin for both datasets is 37. Deviating from this value affects the test accuracy. Additionally, we tracked the average distance in positive pairs and negative pairs in the training set. After training, the average distances for positive pairs were 6.2 and 6.8 in the training sets of the Utah and TCGA ccRCC datasets, respectively. The average distances for negative pairs were 46.5 and 45.6 in the Utah and TCGA ccRCC datasets, respectively.

In common self-supervised learning models such as SimCLR (Chen et al., 2020a) and MoCo (He et al., 2020), projection heads are usually used between the encoders’ outputs and the contrastive loss calculation. We also explored the possibility of incorporating projection heads in our semi-supervised settings. We used the same projection head structures as the default settings in MoCo v3 (Chen et al., 2021). As demonstrated in Table 3, incorporating projection heads resulted in 0.80% and 1.93% reductions in accuracy in two datasets. A potential explanation is that our contrastive learning setup involves the H channel and E channel, which resemble multi-modal data. Additionally, Models fine-tuned for specific tasks sometimes skip the projection heads, assuming the backbone embeddings are sufficient and optimizing directly for task performance. For instance, CADA-VAE (Schonfeld et al., 2019), which is a task-specific model designed for multi-modal inputs, does not incorporate a separate projection head. Instead, its encoders directly produce latent representations. As a result, we did not incorporate projection heads in our model.

InfoNCE loss (Oord et al., 2018) is a commonly used contrastive loss structure in self-supervised learning methods (Chen et al., 2020a; He et al., 2020). In this subsection, the InfoNCE loss is also evaluated. In experiments, we replaced our triplet loss with the InfoNCE loss by referring to the InfoNCE loss implementation from Chen et al. (2021). Table 3 presents the experiment results of using InfoNCE as the contrastive loss function. As shown, our best model with original Triplet loss outperforms InfoNCE on both the Utah and TCGA tasks. One possible explanation is that our contrastive learning leverages two modalities (Hematoxylin and Eosin) rather than two augmentations of the same original sample, which may enable the learning process to benefit from a simpler contrastive loss term. Another possible explanation is that InfoNCE loss requires a very large batch size for optimum performance (Chen et al., 2020a), whereas our batch size is relatively small in our semi-supervised settings due to the limitation of our computational resources.

## 5. Conclusion

In this paper, we proposed a novel semi-supervised model named CLASS-M for histopathological image classification by introducing adaptive stain separation-based contrastive learning and adopting pseudo-labeling using MixUp. We provided the newly annotated Utah ccRCC dataset and TCGA ccRCC dataset. Experiments have shown that our CLASS-M model consistently reached the best classification results compared to other state-of-the-art general semi-supervised and self-supervised computer vision models. Our model demonstrates the capability to perform accurate patch-level classification at various resolutions with only rough annotations on approximately 30 WSIs in training. The code for our model is also publicly available.

The advantage of semi-supervised learning is the end-to-end training despite the sacrifice of convenience compared to self-supervised learning. Self-supervised learning freezes the pre-trained encoders in final training with labeled data, which lacks the flexibility to fine-tune the whole model.

Future work may involve addressing the challenge of handling noisy labels. In our datasets, there is a small portion of tiles that contain only blood vessels, which makes labels inaccurate. A model with more capabilities to tolerate noisy labels may be an interesting road to explore. Our method can also be readily applied to other types of histopathological images, such as immunohistochemistry (IHC) stained images.

## Figures and Tables

**Table 1:** Performance of different classification models on the Utah ccRCC dataset and the TCGA ccRCC dataset. Mean accuracy and standard deviation on the test set are calculated. The supervised setting was only trained on labeled training samples. The encoders of self-supervised learning models, including Barlow Twins, SwAV, MoCo v3, and ViT-DINO were pre-trained on both labeled and unlabeled training samples. The results with the best mean accuracy are shown in bold.

	Models	Test accuracy (Utah)	Test accuracy (TCGA)

Supervised (labeled images only)	ResNet	88.85 + 2.66%	72.11 ± 0.41%
	ViT	84.69 ± 1.33%	73.50 ± 0.94%

Self-supervised (pre-trained on labeled and unlabeled images)	Barlow Twins	93.42 ± 0.43%	77.42 ± 4.92%
	SwAV	93.87 ± 0.59%	82.17 ± 0.05%
	MoCo v3	93.91 ± 0.60%	78.82 ± 0.93%
	ViT-DINO	90.53 ± 1.13%	79.76 ± 2.15%

Semi-supervised (trained on labeled and unlabeled images)	FixMatch	91.58 ± 0.65%	83.34 ± 2.53%
	MixMatch	92.94 ± 1.54%	88.35 ± 1.39%
	CLASS (ours)	94.92 ± 0.67%	83.06 ± 0.47%
	CLASS-M (ours)	**95.35 ± 0.46%**	**92.13 ± 0.89%**

**Table 2:** Ablation studies of CLASS-M models on the Utah ccRCC dataset and the TCGA ccRCC dataset. Mean accuracy and standard deviation on test set are calculated. The results with best mean accuracy are shown in bold.

Models	Test accuracy (Utah)	Test accuracy (TCGA)

CLASS-M	**95.35 ± 0.46%**	**92.13 ± 0.89%**
CLASS-M without contrastive loss	90.92 ± 1.18%	89.70 ± 0.77%
CLASS-M without aug on RGB images	94.41 ± 0.44%	91.21 ± 2.05%
CLASS-M without adaptive stain separation (use fixed stain separation)	93.97 ± 0.40%	90.97 ± 1.86%
CLASS	94.92 ± 0.67%	83.06 ± 0.47%
CLASS (use Red/Green channels as two views)	90.75 ± 0.13%	81.14 ± 0.34%
CLASS (use Red/Blue channels as two views)	89.06 ± 0.54%	80.14 ± 2.66%
CLASS (use Green/Blue channels as two views)	83.43 ± 3.63%	80.25 ± 1.15%
CLASS without contrastive loss	84.57 ± 2.40%	74.46 ± 2.03%
CLASS without aug on RGB images	94.99 ± 0.58%	82.78 ± 1.10%
CLASS without adaptive stain separation (use fixed stain separation)	92.43 ± 0.34%	79.89 ± 0.69%
CLASS without aug on RGB images and adaptive stain separation	91.53 ± 1.52%	75.67 ± 1.76%

**Table 3:** Extended experiments of contrastive loss for CLASS-M models on the Utah ccRCC dataset and the TCGA ccRCC dataset. Mean accuracy and standard deviation on the test set are calculated. The results with the best mean accuracy are shown in bold.

Losses	Test accuracy (Utah)	Test accuracy (TCGA)

Triplet loss (margin = 32)	93.51 ± 0.57%	90.73 ± 1.61%
Triplet loss (margin = 35)	94.43 ± 0.71%	91.67 ± 0.87%
Triplet loss (margin = 37)	**95.35 ± 0.46%**	**92.13 ± 0.89%**
Triplet loss (margin = 40)	94.24 ± 0.19%	91.36 ± 0.85%
Triplet loss (margin = 45)	93.12 ± 0.55%	90.96 ± 0.51%
Triplet loss (margin = 37) with projection heads	94.55 ± 1.03%	90.20 ± 0.29%
InfoNCE loss	92.15 ± 0.90%	90.75 ± 0.66%
InfoNCE loss with projection heads	91.85 ± 1.47%	91.75 ± 1.15%

**Fig. 5.**
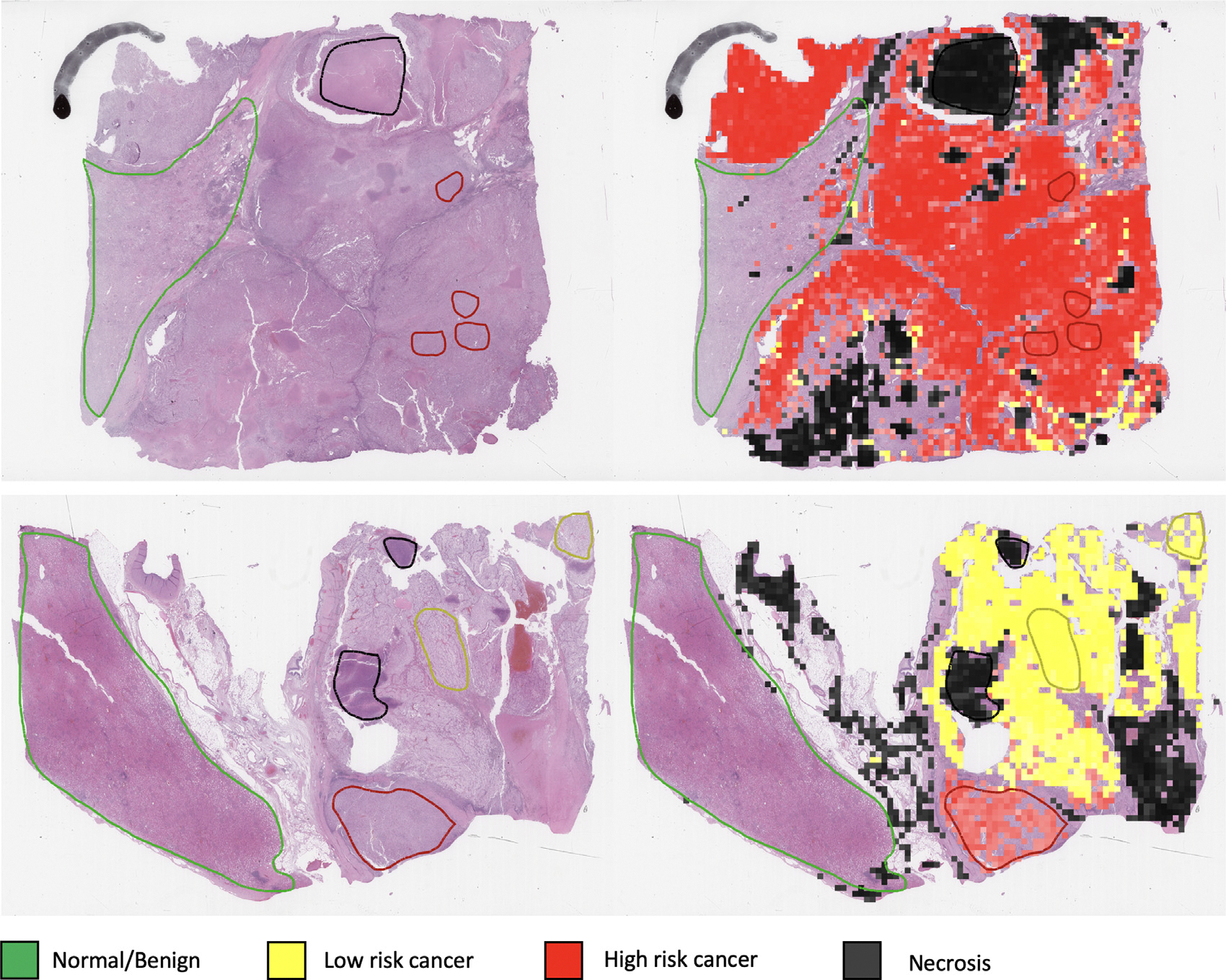
Visualization of our CLASS-M model’s predictions on the Utah ccRCC WSIs from test set. The polygons show the annotations on WSIs. (Green: Normal/Benign, Yellow: Low risk cancer, Red: High risk cancer, Dark Grey: Necrosis) Each polygon labeled as low/high risk cancer marks the region of a certain cancer growth pattern. The heatmaps show the model’s predictions on all foreground tiles when the predictions are not Normal/Benign. The strength of color shows the confidence of prediction.

**Fig. 6.**
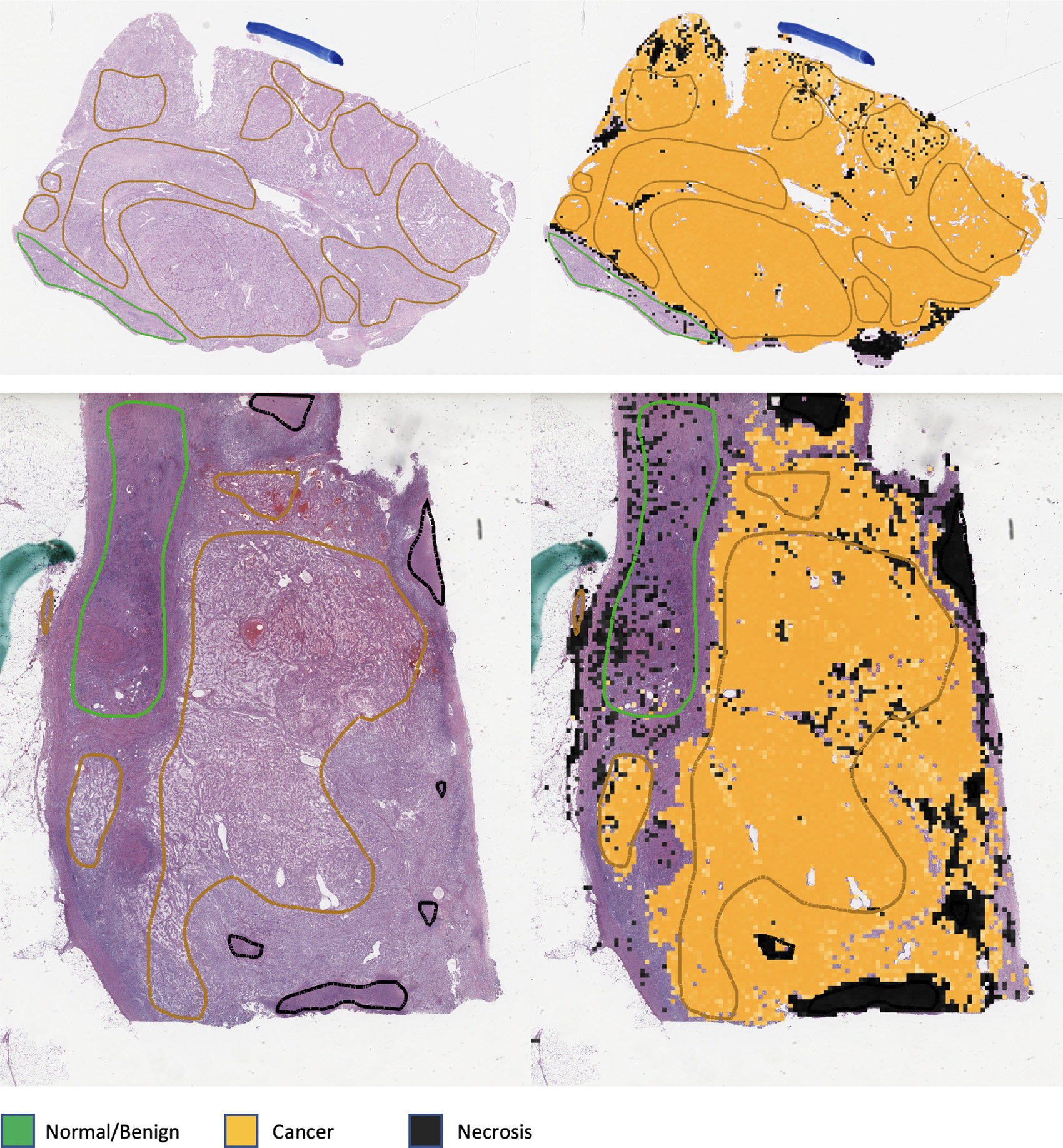
Visualization of our CLASS-M model’s predictions on the TCGA ccRCC WSIs from the test set. The polygons show the annotations on WSIs. (Green: Normal/Benign, Orange: Cancer, Dark Grey: Necrosis) Each polygon labeled as cancer marks the region of a certain cancer growth pattern. The heatmaps show the model’s predictions on all foreground tiles when the predictions are not Normal/Benign. The strength of color shows the confidence of prediction.

## Data Availability

Our related research data and code is publicly available at https://github.com/BzhangURU/Paper_CLASS-M/tree/main.

## Footnotes

2 The Utah ccRCC dataset is available through a transfer agreement by contacting the authors.

## Appendix A. More details on experiment results

Please check Tables 4 and 5 for recall, precision and F-score of each model on the Utah ccRCC dataset and the TCGA ccRCC dataset.

## Appendix B. Visualization of our CLASS-M model predictions

Please check Figs. 5 and 6 for visualization of our CLASS-M model’s patch-level predictions on WSIs from test set for both datasets.

## Appendix C. Pseudo-labeling accuracy on the TCGA ccRCC dataset

In the TCGA ccRCC dataset, only 30 out of 300 training slides provide labels in our semi-supervised settings. Among the remaining 270 training slides, we provide additional polygon labels for 4 slides. The unlabeled training tiles in our CLASS-M experiments that fall inside these extra polygons are used to evaluate the accuracy of the generated pseudo-labels. In summary, there are 3986 Normal/Benign tiles, 6329 Cancer tiles, and 71 Necrosis tiles. The average pseudo-labeling accuracy in each category across three runs is as follows: 98.59% for Normal/Benign tiles, 94.56% for Cancer tiles, and 84.04% for Necrosis tiles. Overall, the average pseudo-labeling accuracy on those 10,386 tiles across three runs is 96.03%.

**Table 4:** Recall, precision and F-score of different classification models on the Utah ccRCC dataset. Mean and standard deviation on test set are calculated based on 3 runs. Green numbers show supervised learning results, blue numbers show self-supervised learning results, and black numbers show semi-supervised learning results.

	Models	Normal/Benign	Low risk cancer	High risk cancer	Necrosis	Average (all classes)

Recall	ResNet	0.9308 ± 0.0358	0.9186 ± 0.0254	0.9717 ± 0.0384	0.7330 ± 0.1172	0.8885 ± 0.0266
	ViT	0.8906 ± 0.0138	0.9571 ± 0.0179	0.7112 ± 0.0351	0.8286 ± 0.0278	0.8469 ± 0.0133
	BT	0.9685 ± 0.0030	0.9693 ± 0.0026	0.9169 ± 0.0083	0.8820 ± 0.0097	0.9342 ± 0.0043
	SwAV	0.9413 ± 0.0049	0.9889 ± 0.0010	0.9674 ± 0.0039	0.8571 ± 0.0242	0.9387 ± 0.0059
	MoCo v3	0.9631 ± 0.0068	0.9755 ± 0.0010	0.9306 ± 0.0089	0.8874 ± 0.0082	0.9391 ± 0.0060
	ViT-DINO	0.9017 ± 0.0230	0.9933 ± 0.0033	0.8946 ± 0.0567	0.8315 ± 0.0357	0.9053 ± 0.0113
	FixMatch	0.8322 ± 0.0759	0.9693 ± 0.0459	0.9563 ± 0.0602	0.9051 ± 0.0558	0.9158 ± 0.0065
	MixMatch	0.9004 ± 0.0261	0.9047 ± 0.0420	0.9588 ± 0.0044	0.9538 ± 0.0092	0.9294 ± 0.0154
	CLASS	0.9753 ± 0.0061	0.9660 ± 0.0092	0.9563 ± 0.0051	0.8994 ± 0.0260	0.9492 ± 0.0067
	CLASS-M	0.9252 ± 0.0194	0.9576 ± 0.0174	0.9880 ± 0.0065	0.9430 ± 0.0051	0.9535 ± 0.0046

Precision	ResNet	0.9702 ± 0.0148	0.8665 ± 0.1087	0.4963 ± 0.1317	0.8930 ± 0.0493	0.8065 ± 0.0493
	ViT	0.9832 ± 0.0034	0.5781 ± 0.0642	0.3941 ± 0.0429	0.8649 ± 0.0547	0.7051 ± 0.0132
	BT	0.9924 ± 0.0008	0.9021 ± 0.0100	0.7107 ± 0.0134	0.8666 ± 0.0277	0.8679 ± 0.0076
	SwAV	0.9855 ± 0.0031	0.9589 ± 0.0018	0.6785 ± 0.0070	0.7442 ± 0.0212	0.8418 ± 0.0029
	MoCo v3	0.9860 ± 0.0010	0.9149 ± 0.0132	0.8037 ± 0.0276	0.8271 ± 0.0279	0.8829 ± 0.0171
	ViT-DINO	0.9803 ± 0.0023	0.7384 ± 0.0237	0.6783 ± 0.1003	0.6655 ± 0.0961	0.7657 ± 0.0358
	FixMatch	0.9913 ± 0.0059	0.6529 ± 0.1011	0.3679 ± 0.0784	0.7837 ± 0.1874	0.6990 ± 0.0776
	MixMatch	0.9945 ± 0.0015	0.8400 ± 0.1067	0.4072 ± 0.0615	0.8660 ± 0.0518	0.7770 ± 0.0401
	CLASS	0.9918 ± 0.0026	0.9165 ± 0.0103	0.7164 ± 0.0411	0.9439 ± 0.0119	0.8921 ± 0.0111
	CLASS-M	0.9980 ± 0.0005	0.9263 ± 0.0102	0.7316 ± 0.0048	0.6760 ± 0.0666	0.8330 ± 0.0192

F-score	ResNet	0.9497 ± 0.0137	0.8903 ± 0.0706	0.6483 ± 0.1052	0.8005 ± 0.0641	0.8222 ± 0.0256
	ViT	0.9346 ± 0.0063	0.7192 ± 0.0495	0.5065 ± 0.0400	0.8452 ± 0.0152	0.7513 ± 0.0156
	BT	0.9803 ± 0.0018	0.9345 ± 0.0044	0.8007 ± 0.0079	0.8741 ± 0.0167	0.8974 ± 0.0060
	SwAV	0.9628 ± 0.0011	0.9737 ± 0.0014	0.7976 ± 0.0062	0.7963 ± 0.0020	0.8826 ± 0.0012
	MoCo v3	0.9744 ± 0.0039	0.9442 ± 0.0074	0.8624 ± 0.0197	0.8561 ± 0.0180	0.9093 ± 0.0122
	ViT-DINO	0.9393 ± 0.0132	0.8469 ± 0.0146	0.7662 ± 0.0428	0.7381 ± 0.0721	0.8226 ± 0.0235
	FixMatch	0.9035 ± 0.0415	0.7785 ± 0.0834	0.5263 ± 0.0777	0.8273 ± 0.0878	0.7589 ± 0.0572
	MixMatch	0.9451 ± 0.0137	0.8692 ± 0.0697	0.5699 ± 0.0600	0.9072 ± 0.0281	0.8228 ± 0.0357
	CLASS	0.9835 ± 0.0019	0.9406 ± 0.0093	0.8187 ± 0.0267	0.9208 ± 0.0080	0.9159 ± 0.0044
	CLASS-M	0.9602 ± 0.0106	0.9416 ± 0.0037	0.8407 ± 0.0050	0.7864 ± 0.0479	0.8822 ± 0.0150

**Table 5:** Recall, precision and F-score of different classification models on the TCGA ccRCC dataset. Mean and standard deviation on test set are calculated based on 3 runs. Green numbers show supervised learning results, blue numbers show self-supervised learning results, and black numbers show semi-supervised learning results.

	Models	Normal/Benign	Cancer	Necrosis	Average (all classes)

Recall	ResNet	0.7136 ± 0.0191	0.9032 ± 0.0105	0.5466 ± 0.0172	0.7211 ± 0.0041
	ViT	0.7502 ± 0.0164	0.8986 ± 0.0130	0.5563 ± 0.0242	0.7350 ± 0.0094
	BT	0.8390 ± 0.0322	0.9003 ± 0.0145	0.5833 ± 0.1345	0.7742 ± 0.0492
	SwAV	0.9542 ± 0.0029	0.9714 ± 0.0011	0.5393 ± 0.0029	0.8217 ± 0.0005
	MoCo v3	0.8479 ± 0.0329	0.9559 ± 0.0078	0.5609 ± 0.0030	0.7882 ± 0.0093
	ViT-DINO	0.9222 ± 0.0511	0.9857 ± 0.0047	0.4850 ± 0.0577	0.7976 ± 0.0215
	FixMatch	0.8582 ± 0.0548	0.9565 ± 0.0165	0.6855 ± 0.0664	0.8334 ± 0.0253
	MixMatch	0.9198 ± 0.0334	0.8910 ± 0.0250	0.8397 ± 0.0156	0.8835 ± 0.0139
	CLASS	0.9291 ± 0.0377	0.9580 ± 0.0212	0.6047 ± 0.0260	0.8306 ± 0.0047
	CLASS-M	0.9477 ± 0.0173	0.9496 ± 0.0060	0.8665 ± 0.0106	0.9213 ± 0.0089

Precision	ResNet	0.6168 ± 0.0214	0.9130 ± 0.0048	0.7622 ± 0.0128	0.7640 ± 0.0062
	ViT	0.6281 ± 0.0302	0.9166 ± 0.0035	0.7879 ± 0.0902	0.7776 ± 0.0385
	BT	0.7161 ± 0.0457	0.9398 ± 0.0256	0.6184 ± 0.1002	0.7581 ± 0.0162
	SwAV	0.8597 ± 0.0039	0.9581 ± 0.0011	0.9211 ± 0.0055	0.9130 ± 0.0025
	MoCo v3	0.7902 ± 0.0241	0.9404 ± 0.0100	0.8681 ± 0.0102	0.8662 ± 0.0082
	ViT-DINO	0.9420 ± 0.0244	0.9374 ± 0.0100	0.9210 ± 0.0125	0.9335 ± 0.0068
	FixMatch	0.8651 ± 0.0677	0.9530 ± 0.0151	0.7338 ± 0.1257	0.8506 ± 0.0224
	MixMatch	0.7505 ± 0.0380	0.9939 ± 0.0031	0.5674 ± 0.0380	0.7706 ± 0.0235
	CLASS	0.8727 ± 0.0472	0.9636 ± 0.0108	0.6986 ± 0.1187	0.8449 ± 0.0452
	CLASS-M	0.8592 ± 0.0249	0.9913 ± 0.0037	0.7420 ± 0.0295	0.8641 ± 0.0130

F-score	ResNet	0.6616 ± 0.0183	0.9081 ± 0.0076	0.6365 ± 0.0117	0.7354 ± 0.0051
	ViT	0.6836 ± 0.0222	0.9075 ± 0.0056	0.6503 ± 0.0308	0.7471 ± 0.0192
	BT	0.7718 ± 0.0266	0.9194 ± 0.0098	0.5849 ± 0.0325	0.7587 ± 0.0195
	SwAV	0.9045 ± 0.0009	0.9647 ± 0.0006	0.6803 ± 0.0032	0.8498 ± 0.0014
	MoCo v3	0.8174 ± 0.0050	0.9481 ± 0.0012	0.6814 ± 0.0021	0.8156 ± 0.0022
	ViT-DINO	0.9314 ± 0.0264	0.9609 ± 0.0058	0.6337 ± 0.0463	0.8420 ± 0.0200
	FixMatch	0.8588 ± 0.0120	0.9546 ± 0.0062	0.7016 ± 0.0439	0.8383 ± 0.0104
	MixMatch	0.8260 ± 0.0273	0.9395 ± 0.0132	0.6767 ± 0.0282	0.8141 ± 0.0209
	CLASS	0.8987 ± 0.0084	0.9606 ± 0.0061	0.6435 ± 0.0429	0.8343 ± 0.0186
	CLASS-M	0.9011 ± 0.0150	0.9700 ± 0.0017	0.7993 ± 0.0211	0.8901 ± 0.0109
